# An Experimental Investigation of White Matter Venous Hemodynamics: Basic Physiology and Disruption in Neuroinflammatory Disease

**DOI:** 10.3389/fneur.2020.00476

**Published:** 2020-06-02

**Authors:** Scott C. Kolbe, Sanuji. I. Gajamange, Jon O. Cleary, Trevor J. Kilpatrick

**Affiliations:** ^1^Department of Neuroscience, Central Clinical School, Prahran, VIC, Australia; ^2^Walt and Eliza Hall Institute, Parkville, VIC, Australia; ^3^Department of Radiology, Guy's and St. Thomas' NHS Foundation Trust, London, United Kingdom; ^4^Florey Institute of Neuroscience and Mental Health, Parkville, VIC, Australia

**Keywords:** white matter, cerebral venous system, hemodynamics, neuroinflammation, cerebral veins, multiple sclerosis

## Abstract

The white matter is highly vascularised by the cerebral venous system. In this paper, we describe a unique blood oxygen-level dependent (BOLD) signal within the white matter using functional MRI and spatial independent components analysis. The signal is characterized by a narrow peak frequency band between 0.05 and 0.1 Hz. Hypercapnia, induced transient increases in white matter venous BOLD that disrupted the oscillation indicative of a vasocontractile mechanism. Comparison of the white matter venous BOLD oscillations between 14 healthy subjects and 18 people with perivenular inflammation due to multiple sclerosis (MS), revealed loss of power in the white matter venous BOLD signal in the peak frequency band (patients = 6.70 ± 0.94 dB/Hz vs. controls = 7.64 ± 0.71 dB/Hz; *p* = 0.006). In MS, lower power was associated with greater levels of neuroinflammatory activity (*R* = −0.64, *p* = 0.006). Using a signal modeling technique, we assessed the anatomical distribution of white matter venous BOLD signal abnormalities and detected reduced power in the periventricular white matter, a region of known venous damage in MS. These results demonstrate a novel link between neuroinflammation and vascular physiological dysfunction in the cerebral white matter, and could indicate enduring loss of vascular compliance associated with imperfect repair of blood-brain barrier damage after resolution of acute neuroinflammation.

## Introduction

Multiple sclerosis (MS) is a chronic inflammatory disorder of the central nervous system. The initiating neuroinflammatory events of multiple sclerosis remain unclear, however, the pathological hallmark of the disease involves peripheral immune cell infiltration via the internal cerebral venous system of the white matter (1) upon disruption to the blood-brain-barrier, leading to perivenular demyelination and axonal injury. Imaging studies have shown that a majority of MS patients have perivenular lesions, leading to recent proposals to include the so-called “central vein sign” as an aid for differential diagnosis (2–4).

Despite the relevance of the white matter venous system to multiple sclerosis pathology, relatively few studies have directly studied venous pathology in the multiple sclerosis brain. Pathological studies have reported enduring damage to the venous system in multiple sclerosis after the resolution of acute inflammation (5, 6). Neuroimaging studies have reported reduced density (7–9), caliber (10, 11), and perfusion (12) of the white matter venous system in multiple sclerosis patients. Together, these findings indicate that the influx of peripheral inflammatory cells into the brain via the cerebral venous system leads to enduring anatomical and physiological changes to the vessels. It is conceivable that such pathologies could confer a susceptibility to further acute inflammation or lead to hypo-perfusion, thus exacerbating neuronal injury.

To further investigate physiological changes in the cerebral venous system, we assessed the blood oxygen-level dependent (BOLD) signal in the white matter using resting-state functional MRI. We show that the cerebral venous system is characterized by a unique oscillatory BOLD signal that is disrupted in people with early multiple sclerosis, and that the degree of signal disruption is associated with the degree of neuroinflammation.

## Materials and Methods

### Subjects

Eighteen participants [7 m/9f; mean (SD) age at the time of testing = 42.4 (10.3) yrs] were recruited prospectively between 2008 and 2010 upon presenting with first demyelinating event acute optic neuritis at the Royal Victorian Eye and Ear Hospital as part of a published trial investigating optic nerve imaging during and after acute optic neuritis (13). Scanning for the present study was performed between 3 and 5 years after initial recruitment as part of an extension study. At the time of scanning, all patients had been diagnosed with clinically definite multiple sclerosis based on the 2010 revisions to the McDonald criteria (14). Fourteen control subjects [6 m/9f; mean (SD) age at the time of testing = 32.5 (4.6) yrs] were also recruited. This study was approved by the Royal Victorian Eye and Ear Hospital and Royal Melbourne Hospital Human Research Ethics Committees. All participants provided voluntary written consent in accordance with the Declaration of Helsinki.

### MRI Acquisitions

All MRI scans were performed using a 3 Tesla MRI system (Trio TIM, Siemens, Erlangen, Germany) with a 32-channel receiver head coil. Three MRI sequences were acquired for each subject: (1) A 3D whole brain double inversion recovery sequence for lesion identification (repetition/echo/inversion times = 7,400/324/450 and 3,000 ms; flip angle = 120°; resolution 0.55 × 0.55 × 1.1 mm^3^ sagittal acquisition); (2) A 3D whole brain MPRAGE T1-weighted sequence for volumetric assessments (repetition/echo/inversion time = 1900/2.63/900 ms; flip angle = 9°; resolution 0.8 × 0.8 × 0.8 mm^3^ sagittal acquisition); and (3) A BOLD-weighted echo planar imaging sequence acquired while subjects watched a blank screen with eyes open (repetition/echo times = 1,400/33 ms; flip angle = 85°; resolution 2.5 × 2.5 × 2.5 mm^3^ axial acquisition; number of BOLD measurements = 440). High spatial and temporal resolution was obtained through the use of “multi-band” simultaneous multi-slice echo planar imaging acquisition (3x acceleration) (15) and GRAPPA in-plane acceleration (2x acceleration). In addition, a single-band image was acquired for each subject (no multi-band acceleration but all other parameters same) which, because of its enhanced gray/white matter contrast, was used for registrations between echo planar imaging and T1 anatomical space.

Lesions were delineated on the double inversion recovery images using a semi-automated thresholding technique (16). Intra-cranial, whole brain parenchyma, gray matter and white matter volumes were obtained using Freesurfer (version 5.0.4) and the standard analysis pipeline (17). Freesurfer segmentations were all assessed by the author and subsequently used to calculate brain, gray matter and white matter volumes, normalized to the intra-cranial volume for each subject.

### BOLD MRI Pre-processing and Spatial Independent Components Analysis

BOLD-weighted MRI scans were processed using FSL MELODIC spatial-ICA software (18). The processing pipeline was as follows for each subject. (1) BOLD time-series image data were linearly realigned to correct for head motion and gradient heating, and registered to the single-band image. One patient and one control subject were excluded due to severe head motion (>1 mm inter-scan motion) during BOLD imaging. (2) Data were high pass filtered (pass band > 0.01 Hz) to remove low frequency signal drift due to MRI gradient heating and spatially smoothed using a non-linear edge-detection based algorithm (19) with spatial extent of 5 mm. (3) Spatial ICA was performed on each subject.

The resulting independent component maps were manually inspected and the white matter component was identified for each subject based on two anatomical features: (a) within the white matter, and (b) in close proximity to the lateral ventricles. Potential differences between patients and controls in the spectral qualities of the independent component time-courses (peak power and frequency) were tested for using Student's *t*-tests. Pearson's correlation analyses were used to test for co-variation between peak power and frequency.

**Figure 1:**
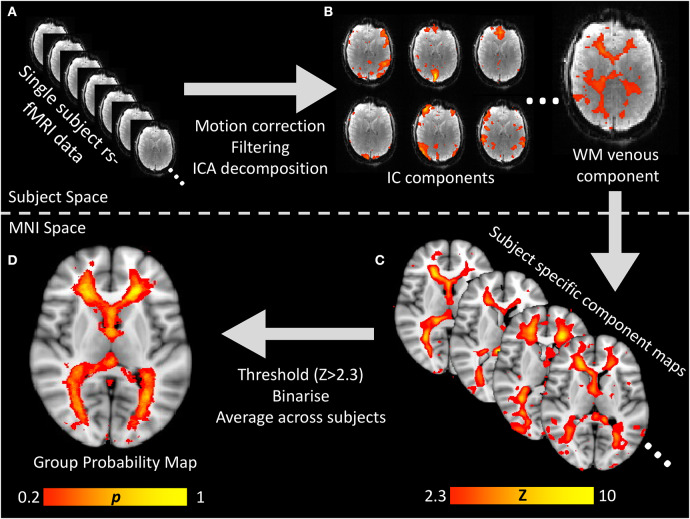
Visual flow chart of the processing pipeline for generating the white matter venous BOLD map. **(A)** Raw resting-state BOLD times series data was preprocessed to correct for head motion, spatially and temporally filtered and then input to spatial ICA decomposition. **(B)** All IC component maps were visualized and the white matter venous component identified based on it's location and power spectral features oscillatory signal with peak power ~0.05 Hz. **(C)** For each subject, the white matter venous component was non-linearly aligned to MNI space, thresholded at Z > 2.3 and binarized. **(D)** All subjects' binarised maps were averaged to create a standard space voxelwise probability map which was used to sample resting-state signals from all subjects for group analyses.

Independent component maps were transformed to standard MNI-152 brain space using a three stage non-linear registration procedure utilizing the Advanced Normalization Tools (ANTs) software (20). (1) Each subject's single-band echo planar imaging reference image was non-linearly registered to the subject's T1 anatomical image. (2) Each subject's T1 anatomical image was registered to the MNI-152 T1 standard brain. (3) The two deformation fields from steps (1) and (2) were added together and the independent component maps were transformed to MNI space using the resulting field. Example venous independent component maps in standard MNI space are shown for two patients and two control subjects in Figure 1A. Potential differences in the anatomical distribution of the IC maps between patients and controls was tested using voxel-wise general linear models and FSL's RANDOMIZE non-parametric permutation sampling statistical tool (21). Resulting statistical maps were corrected for multiple comparisons using the threshold-free cluster enhancement algorithm (22).

To generate a probabilistic standard space map of the white matter veins based on the independent component maps, each subject's standard space independent component map was thresholded (Z > 2.3) and binarised. The resulting binary masks were averaged across all subjects to obtain a map where each voxel value represented the proportion of subjects where that voxel was within the venous independent component map. The entire processing pipeline is summarized in Figure 1.

### Replication Using Human Connectome Project Data

To confirm the spatial and spectral features of the white matter venous BOLD signal, we replicated the previous analyses in an independent dataset of 30 healthy resting state fMRI datasets obtained from the Human Connectome Project (HCP) (23). Thirty random healthy resting-state 3 Tesla fMRI datasets were obtained from the Washington University—University of Minnesota Human Connectome Project (23). Prior to downloading, the datasets had been minimally pre-processed prior to downloading according to the protocol described in Glasser et al. (24) including motion correction and nonlinear registration to MNI-152 atlas space. After downloading we performed spatially smoothed using a non-linear edge-detection based algorithm (19) with spatial extent threshold of 4 mm, and temporally high pass filtered (pass band > 0.01 Hz) prior to single-subject ICA for each dataset. Spatial ICA was performed using FSL MELODIC. The analysis was constrained to obtain only 80 components for each subject to limit the processing time. This number was found to be liberal enough to reliably identify the white matter component. The white matter component was identified in each subject by an experienced observer (SK) in all but three subjects. Data for these three subjects was observed to be high in temporal noise and multi-band artifacts (between slice signal correlations).

## 7Tesla MRI Venography and Bold Imaging

To confirm the venous origin of the white matter IC map, a single healthy volunteer (26 year old female with no history of neurological or vascular disease) was imaged using a 7 Tesla MRI System (Magentom, Siemens, Erlangen). Two sets of images were collected: (1) a gradient echo sequence for calculation of the susceptibility weighted image (repetition/echo times = 24/17.34 ms; flip angle = 13°; resolution 0.75 × 0.75 × 0.75 mm^3^ axial acquisition); and (2) a multi-band BOLD-weighted echo planar imaging sequence with imaging parameters comparable to those used for 3T MRI (repetition/echo times = 1,500/25 ms; flip angle = 44°; resolution = 2 × 2 × 2 mm^3^ axial acquisition; number of BOLD measurements = 205), acquired while subjects watched a blank screen with eyes open.

BOLD data were processed using MELODIC according to the methods described above for 3 Tesla data and linearly registered to the high-resolution gradient echo image using FLIRT (FSL, FMRIB, Oxford, UK).

### White Matter Venous Hypercapnia Challenge

To test the effects of non-neurally driven BOLD signal changes on the white matter venous system, three healthy male subjects (ages = 39/36/31) underwent two runs of functional MRI whilst performing a simple breath hold task to induce transient hypercapnia. The first run consisted of 7 repetitions of 18 s breath hold followed by 24 s normal breathing. The second run consisted of 24 s of breath hold followed by 18 s of normal breathing. BOLD functional MRI data were pre-processed to correct for motion, high-pass and spatially filtered as previously described above. Mixed-effects general linear models were used to test for the following main effects: condition (hypercapnia vs. baseline), tissue type (gray vs. white matter), and duration (short vs. long breath hold), and following interactions: condition/tissue type and condition/tissue type/duration. *Post-hoc* correlation analyses were used to compare BOLD signal changes between tissue types.

### White Matter Venous BOLD Spectral Analysis

In order to compare the power spectra of actual BOLD signal time-courses between patients and controls, rather than IC time-courses, the white matter probability map was used to calculate the weighted-average power-spectrum for all brain voxels for each subject. Briefly, raw BOLD time-course data were pre-processed to correct for head motion and perform high pass filtering. No spatial smoothing was performed. For each voxel, the BOLD time-course was converted to a power spectrum using non-parametric multi-taper spectral decomposition (25) implemented in the MATLAB^®^ R2016a Signal Processing Toolbox (mathworks.com/help/signal/ref/pmtm.html). A weighted mean power spectrum was calculated for each subject using the weighting values from the spatial probability map. The average power within a conservatively judged peak region (0.04 to 0.1 Hz) was compared between patients and control subjects using a Student's *t*-test. Pearson's correlation analyses were performed between average power and logarithmically transformed lesion volume and brain, gray, and white matter fractions.

### White Matter Venous BOLD Voxel Wise Group Comparisons

A data driven signal modeling approach was used to test for putative anatomical differences in the white matter venous BOLD signal between patients and controls. For each brain voxel, high-pass filtered BOLD signals (<0.01 Hz) were converted to power spectra using multi-taper spectral decomposition in MATLAB^®^ R2016a. Each voxel's power spectrum was fitted with a Gaussian curve using nonlinear least squares with appropriate boundary conditions (0.04<μ<0.1 Hz; σ >0 Hz) using MATLAB^®^ R2016a. The fitted power and frequency for the spectral peak was compared voxelwise between groups using a general linear model permutation sampling method (RANDOMIZE, FSL, FMRIB, Oxford, UK) (21) family-wise error corrected with threshold-free cluster enhancement (22).

## Results

### Characterization of the White Matter Venous BOLD Signal in Healthy Subjects

**Figure 2:**
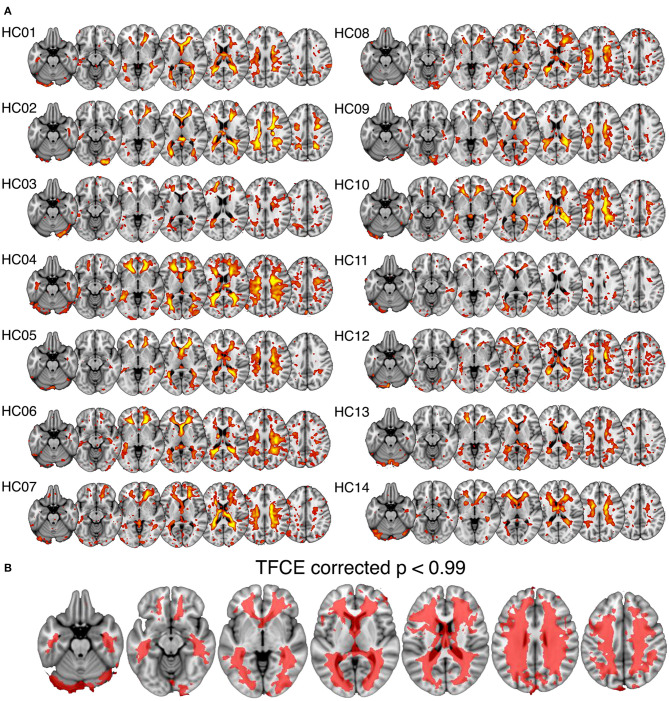
Spatial maps of the white matter venous BOLD signal output from spatial independent components analysis. **(A)** Shows individual component maps for the white matter venous BOLD signal. **(B)** Shows the regions of significant correspondence across subjects using a single group *t*-test, family-wise error corrected using the threshold-free cluster enhancement (TFCE) method.

In all subjects a spatial ICA component was identifiable in the periventricular white matter (Figure 2A). Single sample statistical analysis demonstrated a large region after family wise error correction across most of the cerebral white matter (Figure 2B).

The anatomy of the signal was investigated more precisely in a single subject using high-resolution functional and susceptibility-weighted MRI at 7 Tesla (Supplementary Figure 1). Susceptibility weighted imaging reveals the venous blood vessels within the white matter as regions of high magnetic susceptibility due to the presence of blood. The ICA component was most evident in regions characterized by medium to large periventricular veins.

Power spectral features of the white matter venous BOLD time-series were also assessed. In all subjects, white matter venous BOLD signals were characterized by a narrow peak frequency band was observed in the range of 0.05–0.070 Hz [mean (SD) = 0.061 (0.008) Hz; peak power (SD) = 34.87 (7.20) dB/Hz]. Peak power and frequency were not correlated (*R* = −0.28).

### Replication Analysis of the White Matter Venous BOLD Signal Using HCP Dataset

**Figure 3:**
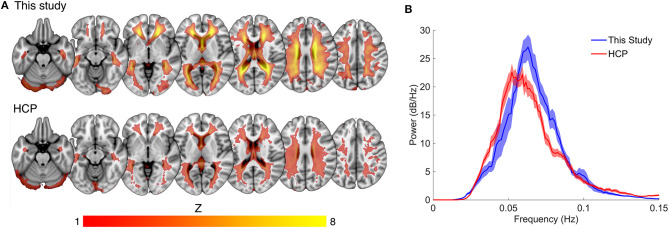
Comparison of spatial and spectral characteristics for the white matter venous BOLD signal between this study the Human Connectome Project (HCP). **(A)** Shows the high degree of spatial congruence between the mean component maps of the two data sets. **(B)** The spectral properties of the white matter venous BOLD signal were similar between the datasets, with both showing a narrow peak high power band between 0.04 and 0.1 Hz.

Compared to the mean white matter IC map for our dataset, the HCP dataset displayed a lower average magnitude (Figure 3A). The same narrow peak frequency band was observed in the HCP data and did not differ significantly in peak frequency or power from our dataset [mean frequency (SD) = 0.057 (0.008) Hz, *Student's t (33 degrees of freedom)* = 1.36, *p* = 0.20; mean peak power (SD) = 32.09 (6.12) dB/Hz, *t*_33_ = 1.33, *p* = 0.21] (Figure 3B). There was no correlation between peak power and frequency (*R* = −0.23).

The white matter component was undetectable in three HCP subjects. To explore this further we compared temporal SNR between our data and the HCP data to determine whether temporal noise could reduce the detectability of the venous component. We observed a relatively large decrease in temporal SNR in the HCP data compared to our data ~60%, despite the large number of extra fMRI volumes collected for each subject (*n* = 400 for our data compared to *n* = 1,200 for HCP data). This difference was likely due to a combination of smaller voxel dimensions (our data = 2.5 mm isotropic, HCP = 2 mm isotropic) and higher multiband acceleration factor (our data = 3, HCP = 5) used by HCP. However, despite noisier and thus less reliable data, the HCP data recapitulated the gross spatial and temporal features of the white matter venous BOLD signal observed in our data.

### White Matter Venous BOLD Physiology During Hypercapnia

Firstly, we observed a significant main effect of condition [*F*_(1948, 1)_ = 53.8, *p* < 0.0001) demonstrating a clear effect of hypercapnia on the BOLD signal. Secondly, we detected a significant interaction between condition and tissue type [*F*_(1948, 1)_ = 1269, *p* < 0.0001] demonstrating that gray matter and white matter responded differently to hypercapnia. *post-hoc* comparisons of BOLD change between gray and white matter showed that while gray matter BOLD reduced as expected (Δ marginal means = −1.50, *p* < 0.0001), white matter venous BOLD signals significantly increased (Δ marginal means = 0.98, *p* < 0.0001). The degree of BOLD increase in the white matter was negatively correlated with gray matter BOLD decreases in all three subjects for both short (subject 1: *R* = −0.44, *p* < 0.0001; subject 2: *R* = −0.73, *p* < 0.0001; subject 3: *R* = −0.50, *p* < 0.0001) and long (subject 1: *R* = −0.35, *p* < 0.0001; subject 2: *R* = −0.63, *p* < 0.0001; subject 3: *R* = −0.46, *p* < 0.0001) breath hold runs (Supplementary Figure 2).

### White Matter Venous BOLD Alterations in Early Multiple Sclerosis

**Figure 4:**
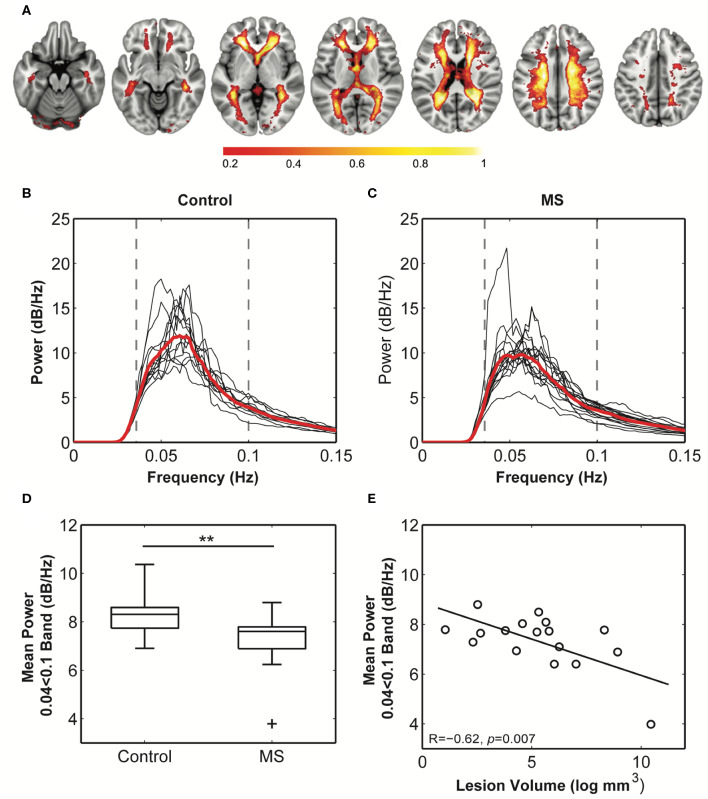
Comparison of the white matter venous BOLD signal between healthy and multiple sclerosis participants. **(A)** The probabilistic map of the BOLD signal across all subjects. Values (0–1) reflect the proportion of subjects with the component in each voxel. Maximal congruence was observed in regions corresponding to the location of the peri-ventricular veins and small veins within the corona radiata. These are also the most common sites of neuro-inflammatory activity in multiple sclerosis. **(B,C)** Weighted average power spectra from within the probability map for control and multiple sclerosis subjects demonstrate a clear peak power within the 0.04–0.1 Hz frequency band. **(D)** Mean power within this band was significantly reduced in multiple sclerosis cases compared to control subjects (^**^ = *p* < 0.01), and **(E)** mean power in patients significantly correlated with logarithmically transformed cerebral lesion volume.

A standard space white matter venous map (Figure 4A) was used to calculate the weighted average raw BOLD signal time-courses for the white matter for each subject (voxels within T2 lesions in patients were omitted in patients). Average white matter venous BOLD power across a conservatively judged peak frequency range (0.04–0.1 Hz) was significantly lower in multiple sclerosis patients (6.70 ± 0.94 dB/Hz, Figure 4C) compared to control subjects [7.64 ± 0.71 dB/Hz, *t*_(28)_ = 2.9, *p* = 0.002, Figure 4B] when compared using a *t*-test (Figure 4D). Variation in the mean power in this band in patients correlated significantly with logarithmically transformed lesion volume (*R* = −0.65, *p* = 0.005; Figure 4E), but not with non-inflammatory markers of brain injury including normalized brain (*R* = 0.22), gray matter (*R* = 0.31), or white matter volumes (*R* = 0.33). These overall statistical results were not affected by narrowing the frequency band used for calculating mean power.

### BOLD Power Spectrum Modeling

**Figure 5:**
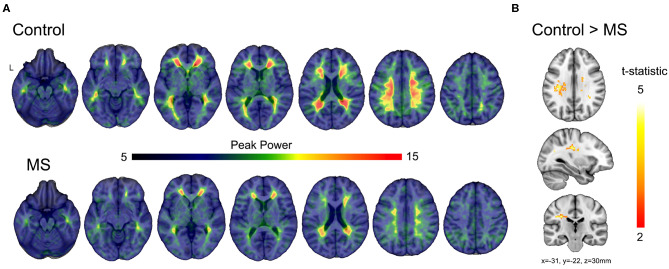
Comparisons of modeled BOLD power between multiple sclerosis and control subjects. **(A)** Average modeled peak power of the BOLD signal for control and multiple sclerosis subjects illustrates a reduction in peak power (dB/Hz) across the much of the white matter. **(B)** The t-statistic map shows regions of significant reduction in peak power in the multiple sclerosis group compared to control subjects after correction using threshold-free cluster enhancement.

In healthy subjects, peak power was constricted to the white matter (Figure 5A upper) demonstrating the specificity of the spectral characteristics to the white matter. Similarly, the multiple sclerosis group showed peak power constrained to the white matter, yet the magnitude of the power was diminished compared to control (Figure 5A lower). Voxel-wise statistical analyses revealed significant loss of power in the periventricular white matter (Figure 5B), consistent with the location of multitudinous small veins that are a common site of inflammatory demyelination in multiple sclerosis.

## Discussion

This study represents, to the best of our knowledge, the first in-depth study of the hemodynamics of the white matter using BOLD-weighted MRI. The white matter venous BOLD signal has been reported previously in the context of removal of artifactual signals from resting state neural connectivity analysis [see Figure 3 from Salimi-Khorshidi et al. (26)]. Perivenular lesions are a consistent feature of multiple sclerosis (27), and the high degree of spatial congruence between the white matter venous BOLD signal and regions of high lesion load in multiple sclerosis (28) led us to explore the signal further as a potential marker for venous pathophysiology. We confirmed that the white matter venous BOLD signal co-localized with smaller and larger internal cerebral venous system using high resolution susceptibility-weighted imaging at 7 Tesla. In our experience, white matter venous BOLD signals are almost always observed in single subject ICA output but are generally not analyzed. Our analyses confirmed that the white matter venous BOLD signal is highly stereotypic across individuals in terms of anatomy and spectral properties. We were also able to confirm spatial and temporal features of white matter venous BOLD in a completely independent dataset (HCP). We expect that the signal could be obtained from any resting-state fMRI data given the reasonably low frequency of the signal (~0.05 Hz) compared to the acquisition frequencies used in most fMRI studies (0.33–1.3 Hz).

To better understand the physiological mechanisms driving the white matter venous BOLD oscillation, we employed dynamic hypercapnic challenge (breath holding) during fMRI scanning in three healthy subjects. Hypercapnia is a potent vasodilator in cerebral gray matter, causing marked BOLD signal attenuation in the cortex commensurate with increased partial volume inclusion of low contrast blood. We therefore expected that hypercapnia induced vasodilation would also affect the white matter venous BOLD signal. In contrast to cortical BOLD, hypercapnia caused a transient increase in the white matter venous BOLD signal the strength of which temporally correlated with the degree of BOLD decrease in cortex. A likely cause for this increase is a reduction in the voxel partial volume inclusion of low contrast venules and veins associated with vasoconstriction. Vasoconstriction in the white matter venous system under hypercapnia could be a mechanism for maintaining intracranial pressure and blood volume during the large vasodilatory response in gray matter regions characteristic of hypercapnia. This is consistent with the well-recognized role of veins as blood capacitance vessels (29).

At rest, the white matter venous BOLD signal was observed to oscillate at around one cycle every 20 s. This rate excludes cardiac and respiratory influences and instead points to a potential auto-regulatory function. Cerebral blood flow is tightly regulated and is resistant to rapid changes in systemic arterial blood pressure (30). The observed BOLD frequency range is within the limits of both myogenic (0.02–0.15 Hz) and sympathetic (0.07–0.15 Hz) vascular regulation (31). In support of a myogenic mechanism, mechanoreceptors have been identified in the walls of the cerebral venous system that are activated by an increase in cerebral blood volume to regulate blood flow (32). The degree of correlation of myogenic regulation across the venous system is unknown. In contrast, sympathetic activity is centrally controlled and known to control vasoconstriction within the cerebral venous system (33). Given the high degree of signal coordination across the entire internal cerebral venous system in healthy individuals, we hypothesize that the observed oscillatory BOLD signal is likely to reflect cyclic vasodilation and constriction under central sympathetic control. Further studies will be required confirm the sympathetic origin of the signal and to elucidate the local control mechanisms.

In multiple sclerosis patients, power in the white matter venous BOLD signal was reduced commensurate with the degree of neuroinflammatory lesion load. This suggests that the physiological substrates of BOLD signal power loss in people with multiple sclerosis relates to inflammatory damage. The white matter venules and veins are the principal entry point for peripheral immune cells into the brain through a disrupted blood-brain barrier during acute inflammation. Such damage to the vascular wall acutely could lead to ongoing postacute damage or imperfect repair. Indeed, previous studies have reported venous atrophy (7–9) and altered blood flow (12) in the white matter of people with multiple sclerosis. Three studies have also reported reduced venous density in multiple sclerosis (7–9), most likely associated with venous atrophy to the point where the blood vessels can no longer be visualized, even with the high resolution (<=0.5 × 0.5 mm^2^ in-plane) afforded by 7 Tesla MRI (8). Consistent with our findings of a link between neuroinflammation and venous damage, Sinnecker et al. (8) showed that venous density was negatively correlated with T2 lesion load. Venous atrophy is a potential substrate for loss of BOLD signal power via the reduction of partial volume inclusion of the venous signal in imaging voxels. Also potentially associated with venous atrophy, reduced venous flow has been reported in the periventricular white matter in both clinically isolated syndromes and relapsing-remitting multiple sclerosis patients using dynamic contrast MRI (12). The authors also observed a non-significant trend toward reduced blood volume, suggestive of vascular atrophy. Gaitán et al. (11) reported narrowing of veins within T2 lesions, yet enlargement of perilesional veins compared to healthy control veins using ultra-high resolution T2^*^-weighted MRI (0.5 × 0.5 × 0.5 mm^3^) during gadolinium infusion. The authors interpreted their findings to indicate that perivascular inflammation could lead to vascular compression and thickening of the perivascular wall. Supporting evidence can be found in histological studies of the vascular system in multiple sclerosis noting fibrinoid and haemosiderin deposition, thrombosis, and venous wall thickening (5, 6). Given the large capacitance of the venous system, it is possible that the perilesional enlargement observed by Gaitán et al. (11) reflects a bottleneck effect caused by flow resistance within lesions, leading to perilesional vascular distension. Together, these studies demonstrate significant neuroinflammatory damage to the white matter venous system that could account for the haemodynamic changes observed in our study. It is conceivable that reduced hemodynamics has the potential to exacerbate neural damage via local ischemia. This hypothesis support further investigation of the white matter venous BOLD signal in the context of other diseases characterized by white matter lesions.

This study has several limitations that should be addressed in follow-up studies. Firstly, we did not directly anatomically image and map the venous system in all subjects, so it was not possible to compare the haemodynamic alterations to venous anatomy and morphology directly. The high resolution afforded by high field (7T+) MRI allows the mapping of fMRI signals to specific blood vessels, and to create maps of the white matter venous system that could be used to better characterize the spatial distribution of venous damage. We did however, exclude voxels from each patient's white matter venous probability map that were classified as lesion on double inversion recovery scans. Therefore, the changes in white matter venous BOLD power observed in patients was measured from normal appearing brain regions that were not influenced by overt signal changes associated with lesion pathology. Finally, our study focused on a convenience sample of patients with a history of acute optic neuritis with a relatively consistent and short disease duration of between 3 and 5 years. Future studies should characterize the progression of white matter haemodynamic abnormalities in later disease stages. Longitudinally designed studies will also be required to determine whether white matter haemodynamic abnormalities are associated with greater susceptibility to subsequent inflammatory cell infiltration or neurodegenerative changes.

## Summary and Conclusions

The internal cerebral veins within the white matter are the most common site of early peripheral immune cell infiltration in the neuroinflammatory demyelinating disease multiple sclerosis. This study identified a novel haemodynamic signal with a narrow spectral peak in the cerebral veins of the white matter using resting-state functional MRI and ICA. Peak power of the signal was reduced in people with multiple sclerosis, and the degree of reduction correlated significantly with neuroinflammatory lesion volume. These results indicate that multiple sclerosis is associated with dysfunction of the white matter veins that initiates early in the disease. Future studies are required to identify the physiological mechanism driving white matter venous BOLD hemodynamics and explore the role of altered hemodynamics in multiple sclerosis pathophysiology.

## Data Availability Statement

Raw anonymised data will be made available upon request to the authors.

## Ethics Statement

The studies involving human participants were reviewed and approved by Royal Victorian Eye and Ear Hospital Human Research Ethics Committee Royal Melbourne Hospital Human Research Ethics Committee. The patients/participants provided their written informed consent to participate in this study.

## Author Contributions

SK conceived of the study, collected and analyzed data and wrote the manuscript. SG made contributions to the interpretation of data for the work and revised the paper critically for important intellectual content. JC made contributions to the interpretation of data for the work and revised the paper critically for important intellectual content. TK made contributions to the interpretation of data for the work and revised the paper critically for important intellectual content.

## Conflict of Interest

The authors declare that the research was conducted in the absence of any commercial or financial relationships that could be construed as a potential conflict of interest.
