# Supplementary material for: An Experimental Investigation of White Matter Venous Hemodynamics: Basic Physiology and Disruption in Neuroinflammatory Disease
Source: Front Neurol. 2020 Jun 2;11:476. doi: 10.3389/fneur.2020.00476 (PMC7280478; doi:10.3389/fneur.2020.00476)
Supplement: Supplementary file 1 [file Data_Sheet_1.docx]

**
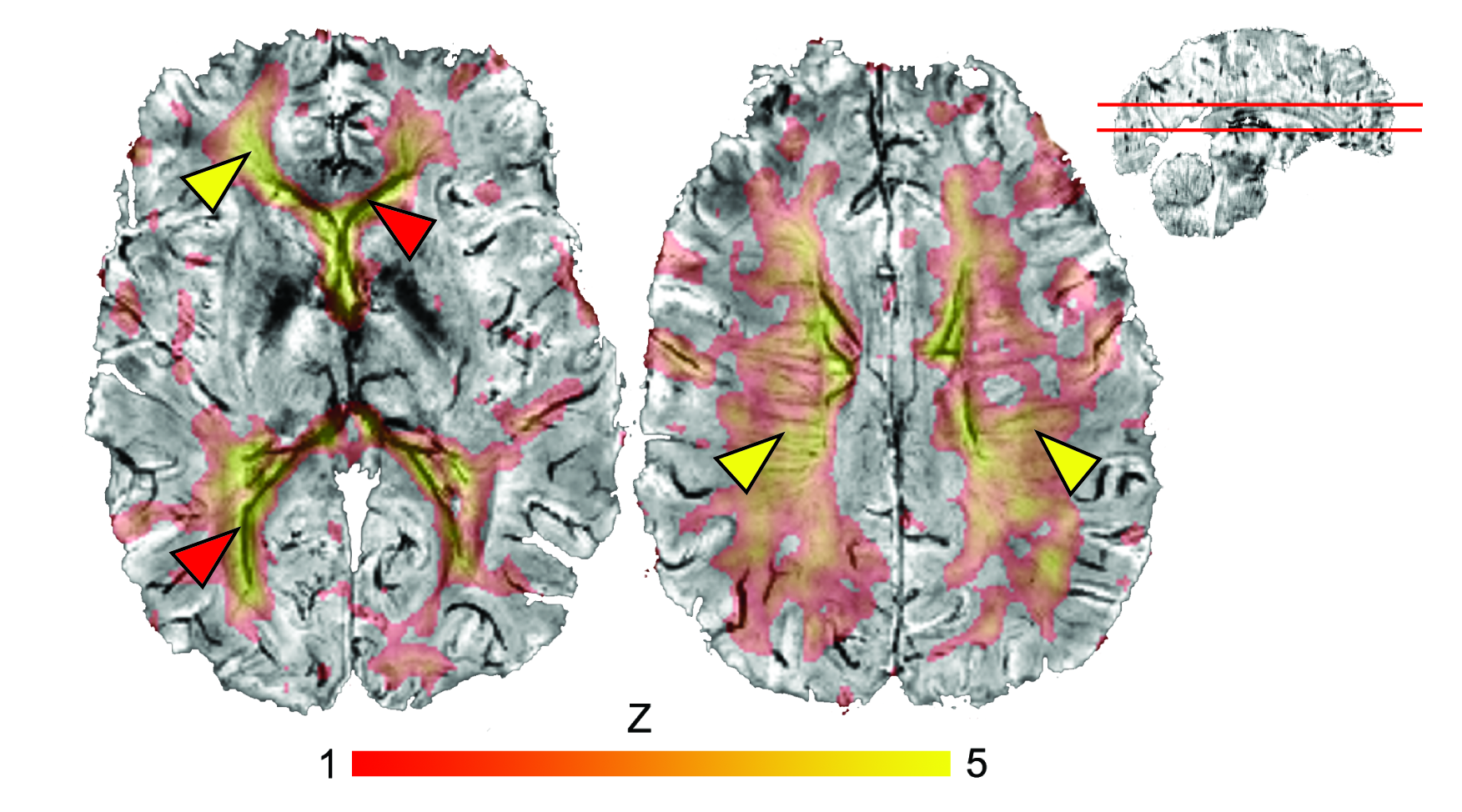
**

***Supplementary Figure 1. Venous BOLD signal ICA output overlaid on the same subject’s susceptibility weighted MRI scan.*** *Veins appear as dark lines in the susceptibility-weighted image, and these co-localise with regions of highest venous BOLD signal in both smaller veins deep in the white matter (yellow arrowheads) and larger periventricular veins (red arrowheads).*

**
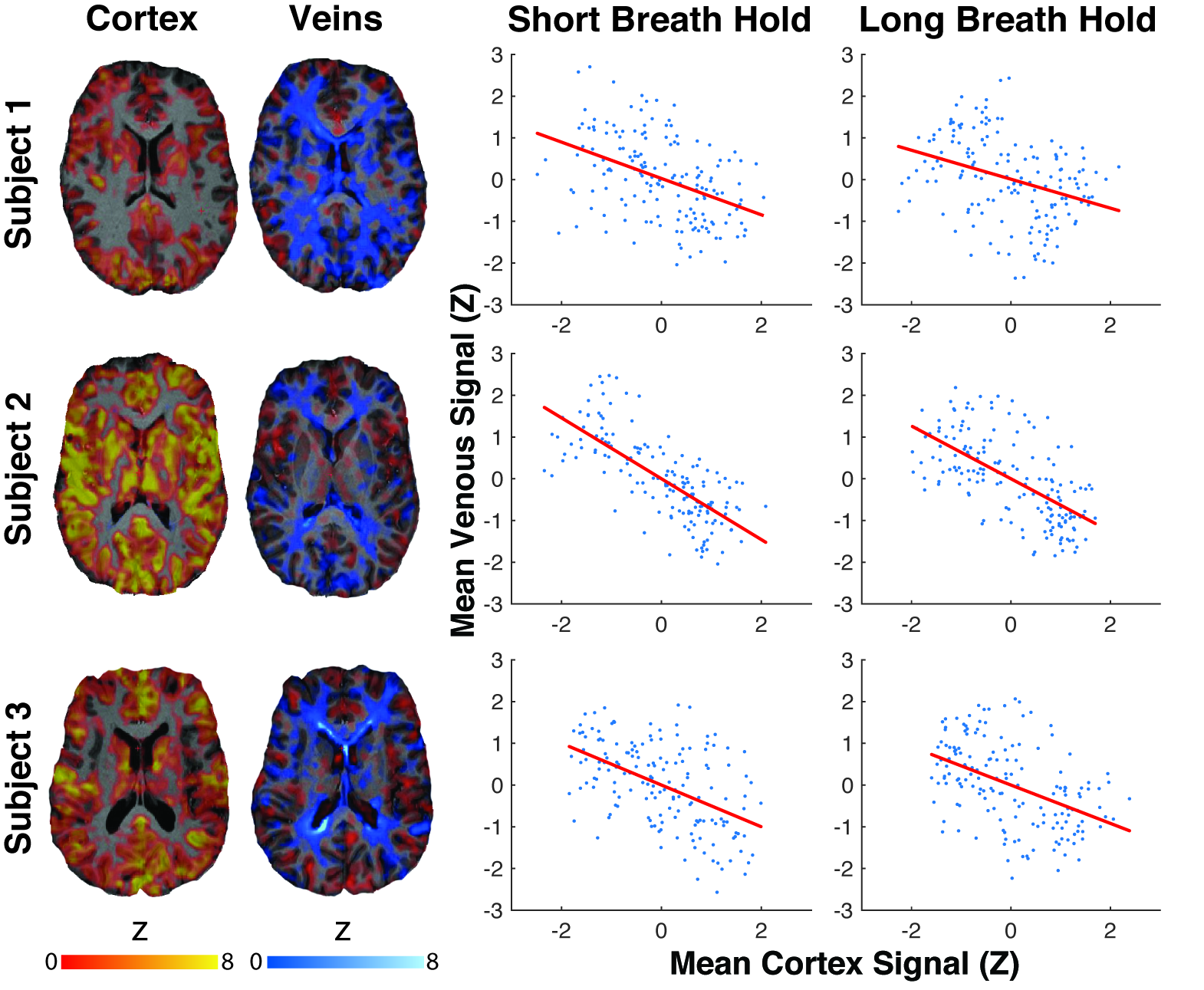
**

***Supplementary Figure 2. Comparison between BOLD signals from cortex and internal cerebral veins during transient hypercapnia*** *(breath hold) in three healthy subjects. The left side shows the spatial maps for cortex and white matter that displayed negative and positive signal changes respectively during breath holds. Signal time course correlations across the entire experiment (right) show that the decrease in cortical BOLD was associated with a contemporaneous increase in venous BOLD.*
